# Experimental Study on the Impact Compression Properties of Aluminum Honeycomb with Gradient-Thickness Cell Walls Using a Three-Factor Orthogonal Matrix Design

**DOI:** 10.3390/ma18163785

**Published:** 2025-08-12

**Authors:** Peng Sun, Xiaoqiong Zhang, Yinghou Jiao, Rongqiang Liu, Tao Wang

**Affiliations:** 1 College of Mechanical and Electrical, Harbin Institute of Technology, Harbin 150001, China; hitsunpeng2013@163.com (P.S.);; 2 Shenzhen CANSINGA Technology Co., Ltd., Shenzhen 518102, China; 3 College of Mechanical Engineering, Taiyuan University of Technology, Taiyuan 030024, China

**Keywords:** honeycombs with gradient-thickness cell wall, impact compression, orthogonal matrix, energy absorption, specific energy absorption

## Abstract

A novel honeycomb with gradient-thickness cell walls (HGTCWs) is fabricated through chemical etching to achieve progressive thickness reduction in the cell walls. This engineered honeycomb demonstrates superior energy absorption by effectively eliminating the peak load during the linear elastic stage of the load–displacement curve under impact loading, thereby preventing premature structural failure caused by excessive instantaneous loads. To systematically investigate the impact compression mechanics, energy absorption characteristics, and key influencing factors of aluminum HGTCWs, a three-factor orthogonal array of low-velocity impact experiments was designed. The design of experimental parameters for the impact test has taken into account the impact mass, impact velocity, and etching height. Comparative analysis assessed how these factors influence energy absorption performance. Results reveal that chemical etching-induced thickness gradient modification effectively suppresses peak load generation. Load–displacement curves exhibit distinct bilinear characteristics: an initial single linear phase when compression displacement is below the etching height, followed by a dual-linear phase with an inflection point at the gradient height. Time–velocity profiles during impact primarily consist of an initial nonlinear deceleration phase followed by a linear deceleration phase. Range analysis and analysis of variance identify impact velocity as the dominant factor influencing the energy absorption characteristics of HGTCWs.

## 1. Introduction

Honeycomb materials have gained extensive applications in aerospace, transportation engineering, and protective equipment due to their high specific strength, superior energy absorption characteristics, and lightweight design [1,2,3]. During service in these critical fields, honeycomb structures are frequently subjected to various impact loads, including high-velocity collisions (e.g., bird strikes on aircraft) and low-velocity impacts (e.g., tool drops during maintenance). Studies have demonstrated that such impacts can significantly degrade structural stiffness and strength [4,5,6]. These findings have motivated substantial research efforts to investigate the impact mechanical responses and energy absorption mechanisms of honeycomb structures.

The failure behavior of honeycomb structures exhibits remarkable complexity, governed by multiple factors including cell geometry, relative density, structural configuration, impact velocity, and manufacturing processes [7,8,9]. Conventional uniform-wall honeycombs as energy absorbers typically demonstrate an initial high instantaneous peak load during compression, followed by load fluctuations before entering periodic plastic folding plateau stages. This dynamic load variation adversely affects both protective performance and energy absorption efficiency. To address this limitation, researchers have proposed structural modifications through surface crease patterns to induce stable plastic folding and mitigate peak loads [10,11]. In early fundamental research on honeycomb materials, researchers focused on the influence of wall thickness distribution on mechanical properties, noting that the solid distribution in edge regions is a critical factor determining the in-plane and out-of-plane elastoplastic behaviors of honeycombs. Compared to traditional uniform-thickness honeycombs, edge-high-density variable-thickness honeycombs exhibit superior compressive buckling strength [12,13]. This finding laid an important foundation for subsequent efforts to enhance honeycomb performance through structural optimization.

With the development of origami technology, researchers began integrating it into innovative honeycomb designs to explore the relationship between geometric deformation and mechanical properties. Ma et al. [14] were the first to apply origami technology to the design of thin-walled tubes, proposing a rigid origami pattern pre-folded into a kite shape as an energy absorption structure. Numerical simulations showed that this design, by guiding the ordered transfer of plastic deformation, increased axial energy absorption efficiency by 29.2% compared to traditional structures while significantly reducing the initial peak load by 56.5%, verifying the potential of origami patterns to regulate structural energy absorption characteristics. Building on this, Albert et al. [15] further developed novel thin-walled square tubes with pre-folded rigid origami patterns on their surfaces. These patterns, acting as “geometric defects,” actively guided structural failure modes, effectively reducing peak loads and providing new ideas for the lightweighting and energy absorption optimization of thin-walled structures.

The successful application of origami technology soon extended to honeycomb materials. Zhai et al. [16] proposed a pre-folded honeycomb energy absorption structure. Analysis of its in-plane compressive performance revealed that the introduction of creases significantly enhanced structural strength—the in-plane strength of the pre-folded honeycomb was nearly eight times higher than that of conventional honeycombs. This breakthrough highlighted the reinforcing effect of origami design on honeycomb mechanical properties. Concurrently, innovations in manufacturing processes provided new dimensions for studying the performance of origami honeycombs: Townsend et al. [17] combined origami technology with 3D printing to fabricate thermoplastic polyurethane honeycombs. By adjusting cell size and wall thickness, they observed significant parameter dependence in structural stiffness, offering experimental evidence for customized honeycomb design.

After the fundamental properties of origami honeycombs were initially revealed, research further deepened toward multifunctional and tunable directions. Li et al. [18] designed programmable two-stage compressive origami metamaterials. By integrating stacked Miura origami and diamond honeycomb structures, they achieved “peak-force-free” uniform deformation under quasi-static compression—this structure dissipates energy in stages, avoiding failure abruptness caused by local buckling in traditional honeycombs while maintaining overall density uniformity, thus providing an innovative paradigm for high-reliability energy absorption structures. Building on this, Li et al. [19] proposed an origami-inspired periodic cellular structure characterized by three-way deformation capability and gradient design: it exhibits the highest load-bearing capacity along the z-axis, while the introduced gradient significantly enhances energy absorption efficiency, verifying the regulatory role of topological design in multi-physical field properties.

Regarding the dynamic failure behavior of origami honeycombs, Zhai et al. [20] further explored the out-of-plane crushing performance of pre-folded honeycombs. They found that the guiding effect of creases stabilizes the folding process—compared to non-creased honeycombs, pre-folded structures unfold plastic deformation in a more orderly fashion under out-of-plane impacts, reducing the risk of debris ejection and providing safer design options for impact protection scenarios. Finally, the integration of manufacturing processes and topological design has emerged as a new research focus. Wang et al. [21] used fused deposition modeling technology to fabricate customizable honeycombs with buckling characteristics. Through out-of-plane compression tests, they revealed topological dependence: increasing the number of layers or arranging asymmetric layers effectively reduces the initial peak load and suppresses fluctuations in mechanical responses, providing guidance for the engineering application of 3D-printed honeycombs in terms of process–performance relationships.

In summary, from early fundamental studies on wall thickness effects to innovative applications of origami technology, and further to the design and fabrication of multifunctional and tunable honeycombs, related research has progressively deepened the understanding of honeycomb material mechanical behaviors, providing comprehensive support from theory to process for the design of lightweight, high-energy-absorption structures.

Driven by the complexity of porous material structures and the high cost of experiments, related theories on the dynamic characteristic analysis of honeycomb structures have developed rapidly. Among them, the most common is the homogenization algorithm, which covers the full scenarios from static to high strain rates and from homogeneous to heterogeneous materials. It can be used to predict key properties such as energy absorption, stress wave propagation, and failure modes. The equivalent single-layer method simplifies complex multi-layer honeycomb structures into an equivalent single-layer continuous medium by applying averaging or homogenization treatment to cell-scale details, thereby reducing computational complexity [22]. The energy equivalent method focuses on energy absorption prediction; periodic/asymptotic expansion methods are suitable for linear/nonlinear dynamic responses; and multi-scale finite element and inhomogeneous inversion methods have promoted the optimization design of complex honeycombs [23,24,25,26,27,28]. In terms of research on the vibration dynamic characteristics of honeycomb structures, Mohammad et al. and Hossein et al. [29,30] adopted a new-type quasi-three-dimensional hyperbolic shear deformation theory, combined with Hamilton’s principle and modified couple stress theory. Through parametric studies, they revealed the influences of porous structure performance, scale parameters, fundamental parameters, and honeycomb geometry on vibration responses. In practical engineering applications, appropriate algorithms should be selected based on dynamic scenarios and honeycomb characteristics, and combined with experimental verification to improve prediction accuracy.

Current research on enhancing energy absorption and mitigating peak loads predominantly focuses on cellular geometry optimization and folding mechanism design. Notably, systematic investigations into axially graded wall thickness honeycombs, particularly those with linear thickness reduction along the height dimension, remain underexplored. To address the limitations identified in prior research, this study proposes a novel chemical etching process to fabricate aluminum HGTCWs. The proposed gradient cell wall thickness design enables the etched regions to yield and absorb energy preferentially, followed by plastic deformation in the unetched regions, forming a two-stage energy absorption zone mode. Additionally, this design effectively suppresses the high overload phenomenon in traditional homogeneous honeycombs during impact, which is more conducive to protecting the safety of structures or personnel. The weight of the material is also further reduced, which is more favorable for lightweighting.

Furthermore, previous experimental studies on aluminum honeycombs have primarily focused on uniform parameters such as cell size and cell wall thickness. In contrast, this study conducted a three-factor orthogonal experimental investigation with impact velocity, impact mass, and etching height as the main variables. It systematically investigated the impact compression performance characteristics of HGTCWs with different etching heights under various impact conditions, as well as the influence laws of different factors on the three performance indicators of energy absorption, specific energy absorption, and plastic deformation of HGTCWs. This expanded research on variables breaks through the limitations of uniform structures and better aligns with the practical needs in engineering to regulate energy absorption characteristics through variable wall thickness.

## 2. Materials and Methods

### 2.1. Experimental Materials and Preparing Technology

**Figure 1:**
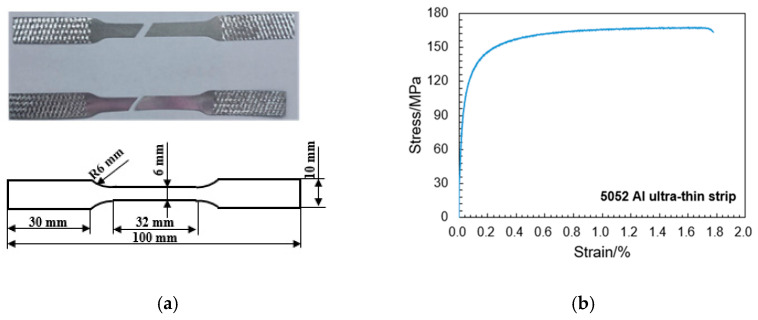
Tensile mechanical property of 5052 Al ultra-thin strip. (**a**) Tensile specimen dimensions and fracture surface. (**b**) Tensile stress–strain curve of 5052 Al ultra-thin foil.

All HGTCW specimens investigated in this study were fabricated using 0.072 mm-thick 5052 aluminum alloy foil as the base material. The base material exhibits a density of 2.68 g/cm^3^, with its chemical composition detailed in Table 1. A quasi-static tensile test was conducted on the 5052 aluminum alloy ultra-thin foil at room temperature using an Instron 68FM-100 material testing machine (Instron Corporation, Norwood City, MA, USA) with the crosshead displacement rate set to 2 mm/min. To prevent the clamp from damaging the 5052 aluminum alloy ultra-thin foil, the clamping ends of the specimen were wrapped with sandpaper to protect the specimen and increase friction. Detailed dimensions of the specimen and the tensile fracture surface are shown in Figure 1a, where the fracture location is in the central region of the gauge length section of the specimen, confirming that the test data are authentic and valid. The quasi-static tensile test reveals that the 5052 aluminum ultra-thin foil exhibits a tensile strength of 167 MPa and a fracture strain of 1.8%, with the stress–strain curve displayed in Figure 1. All non-etched honeycombs with uniform cell walls (HUCWs) and HGTCW specimens were manufactured by Shenzhen CANSINGA Technology Co., Ltd. (Shenzhen, China)

The fabrication of HGTCWs primarily involves chemically etching the cell walls of uniform-thickness honeycomb cores (HUCWs) in an acidic solution. The specific procedure comprises the following steps: (1) prepare multiple liters of dilute hydrochloric acid solution (10% concentration) and distribute it into three plastic containers at solution depths of 50 mm, 100 mm, and 150 mm, respectively; (2) clean HUCW specimens with acetone or ethanol to remove surface contaminants and oil residues, followed by air-drying; (3) immerse the HUCW specimens in the hydrochloric acid solution, securing it with a top-mounted fixing clamp to enable controlled vertical extraction at constant rates. Varying the withdrawal speed regulates the etching duration across the cell walls.

**Figure 2:**
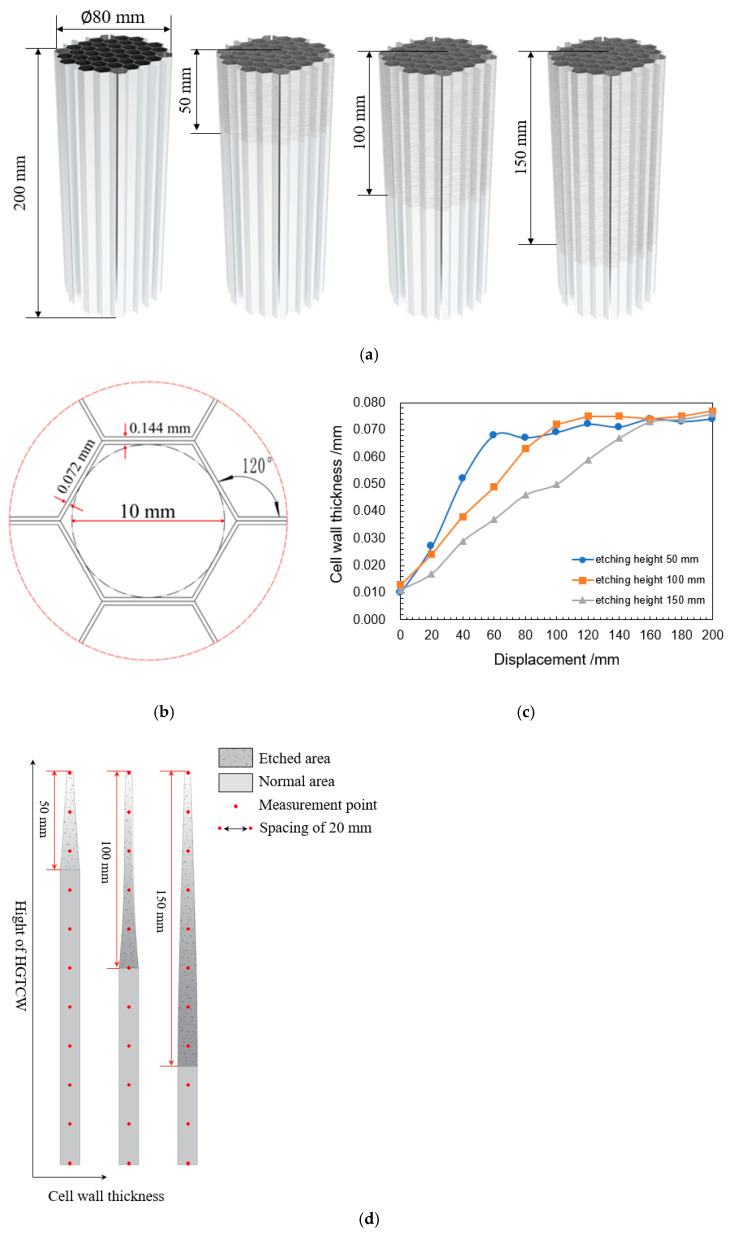
HGTCW appearance structure, dimensions, and cell wall thickness. (**a**) Macro morphology and etching height. (**b**) Honeycomb configuration and dimensions. (**c**) The thickness change of etched honeycomb specimen in the gradient-thickness cell wall region. (**d**) Cross-sectional schematic diagram of the cell wall of HGTCWs.

**Figure 3:**
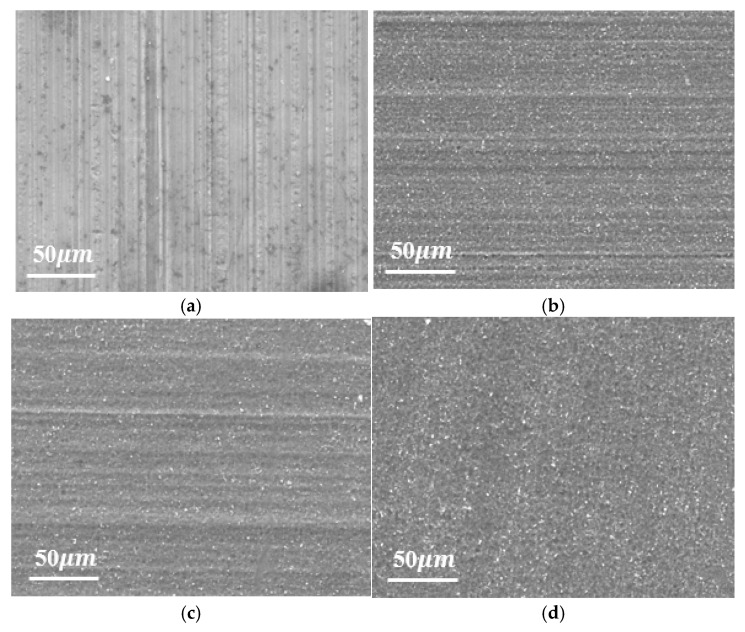
Surface morphology of aluminum foil at different positions of HGTCWs with 150 mm etching height. (**a**) Unetched area. (**b**) Lightly etched area. (**c**) Moderately etched area. (**d**) Heavily etched area.

To investigate etching height effects on HGTCW’s energy absorption performance, three specimen groups with etching heights of 50 mm, 100 mm, and 150 mm were fabricated using a constant 100 min etching duration, as illustrated in Figure 2a. Figure 2b details the cellular geometry dimensions. During the vertical withdrawal process, the base region experiences maximum etching exposure due to prolonged acid contact, while the top region undergoes minimal exposure. This differential etching produces progressive linear wall thickness reduction along the honeycomb height direction. To verify linear thickness gradation, micrometer measurements were conducted at 20 mm intervals along the HGTCW height. The resulting thickness variation profile (Figure 2c,d) demonstrates near-linear thickness progression in the etched region; this process can achieve a certain degree of control over the thinning of aluminum honeycomb wall thickness. To further examine the etching effects of the acidic solution etching process on the aluminum foil surface, Figure 3a–d present surface micrographs sampled at 50 mm intervals along the 150 mm etched height specimen. Figure 3a depicts the unetched Al foil region, revealing characteristic drawing-induced micro-indentations from the manufacturing process. In Figure 3b–d, progressive etching duration correlates with (1) gradual elimination of initial surface indentations, (2) increased surface roughness, and (3) development of uniformly distributed micro-pits. These observations confirm that controlled etching with appropriate acid concentration and duration achieves cell wall thickness reduction without inducing perforation defects, while maintaining high surface quality in aluminum honeycombs.

Prior to testing, each specimen was weighed using a precision electronic balance (resolution: 0.01 g, Leqi Electronic Technology Co., Ltd., Dongguan, China). The average masses of specimens with gradient heights of 50 mm, 100 mm, and 150 mm were measured as 47.41 ± 0.15 g, 43.02 ± 0.12 g, and 38.35 ± 0.18 g, respectively, confirming mass consistency within experimental tolerances.

### 2.2. Low-Velocity Impact Testing

**Figure 4:**
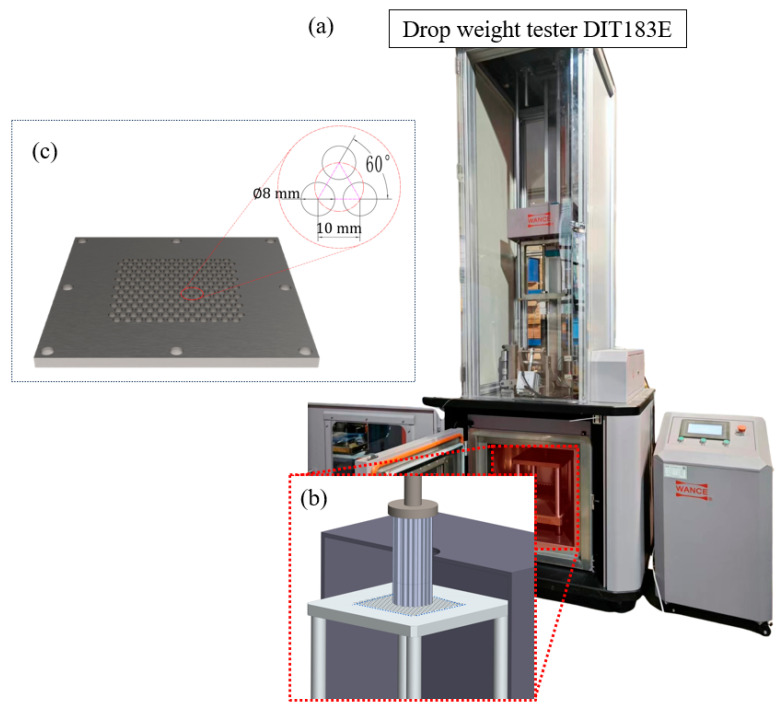
Drop-weight test system. (**a**) DIT183E drop-weight test machine. (**b**) Impact pressure plate and honeycomb specimen. (**c**) Porous plate.

The low-velocity impact experiments were conducted using a WANCE-DIT183E drop-weight impact testing system (Shenzhen Wance Test Equipment Co., Ltd., Shenzhen, China), equipped with a cylindrical steel impactor (diameter: 100 mm, thickness: 25 mm) as illustrated in Figure 4a. The impact energy was systematically modulated through calibrated mass blocks, achieving three distinct striker masses, 15 kg, 30 kg, and 45 kg, in accordance with experimental design requirements. Furthermore, the integrated software system of the domestically produced DIT183E drop-weight machine used in this experiment enables direct input of counterweight mass and target impact velocity. Prior to formal testing, the hammer is initially lowered to establish positioning contact with the specimen surface. Subsequently, the software automatically calculates the required drop height, elevates the hammer to the computed position, and executes the impact event, thereby guaranteeing precise achievement of the designated impact velocity.

During dynamic compression, gas entrapment within honeycomb cells significantly influences mechanical responses through multiphase interactions [32]. Rapid cell compression generates localized high-pressure gas domains, inducing transverse stresses on cell walls analogous to pneumatic inflation effects. This phenomenon may trigger premature wall buckling or fracture. To eliminate such gas-mediated artifacts, a perforated backing plate with hexagonal pore arrays matching the honeycomb topology was designed (Figure 4c). In this study, the diameter of the inner inscribed circle of the honeycomb cell is 10 mm; therefore, the distance between the centers of adjacent cells is also 10 mm. The diameter of the circular holes in the perforated plate was designed to be 8 mm, with a center-to-center distance of 10 mm between adjacent holes. During specimen placement, marking for positioning was used to ensure that the center of the inner inscribed circle of the specimen’s honeycomb cell aligned with the center of the circular hole in the porous plate. This design effectively and promptly expels air inside the cells without altering the contact between the honeycomb specimen and the supporting plate.

A piezoelectric load cell (sampling rate: 1 MHz) integrated with the impactor recorded real-time force signals. Impact displacement was derived from the force–time profile using the double-integration method expressed in Equation (1) [33]:

(1) $$x\left(t\right)=x_{i}+v_{i}t+\frac{\left(gt^{2}\right)}{2}-\int_{0}^{t}\left(\int_{0}^{t}\frac{F\left(t\right)}{m}d\tau \right)d\tau$$

Within this kinematic framework, $x_{i}$ (initial displacement) and $v_{i}$ (initial velocity) represent the kinematic state variables at t=0, corresponding to the impact initiation moment. F(t) denotes the time-dependent load profile captured by the piezoelectric transducer. m constitutes the total inertial mass of the impact system, comprising both hammer and added counterweights.

### 2.3. Quasi-Static Compression Characteristics of HUCWs

**Figure 5:**
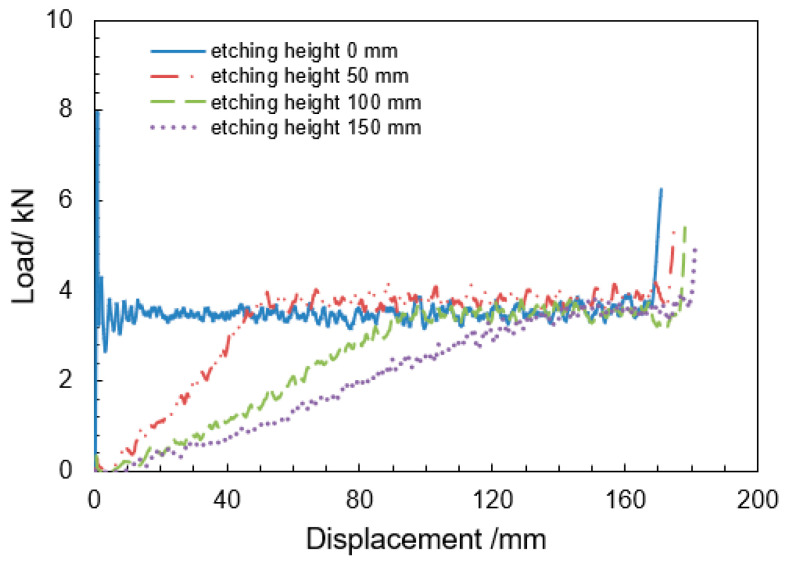
Quasi-static compressive load–displacement curves of HUCWs and HGTCWs.

Quasi-static compression tests were conducted using an Instron 5969 universal testing system at a constant crosshead speed of 2 mm/s. Static compression tests were conducted on the HUCWs and the three types of HGTCWs presented in Figure 2a to preliminarily characterize their mechanical properties. The resultant load–displacement curves (Figure 5) reveal distinct deformation regimes.

For HUCWs: (1) elastic deformation stage: linear response with initial peak load of 8.02 ± 0.15 kN; (2) yield plateau stage: post-yield load stabilization at 3.48 ± 0.12 kN (230% lower than peak load); (3) densification stage: rapid load increase due to cell wall contact.

HGTCWs exhibited the following three critical behavioral divergences: (1) stiffness reduction: initial stiffness (calculated via Equation (2)) decreased from 0.073 kN/mm to 0.024 kN/mm with increasing etching height; (2) peak load elimination: smooth load transition to plateau regime without stress overshoot; (3) yield point delay: yield initiation displacement correlated positively with etching height.

These results demonstrate that HGTCWs enable controlled sequential collapse for progressive energy dissipation, with deformation localization exhibiting strong design dependency on etching parameters. The engineering stiffness was quantified using the standardized formula:

(2) $$k=\frac{∆F}{∆\left(∆L\right)}$$

Among them, k represents the structural stiffness, ∆F represents the load increment, and ∆(∆L) represents the displacement increment.

### 2.4. Three-Factor Orthogonal Array Experimental Design

The three-factor matrix experiment is a common experimental design method [34], primarily used to study the effects of three independent variables (referred to as “factors”) on a response variable, while considering all possible factor combinations and their interactions. In the experiment, each factor can be set to multiple levels, and all combinations of factor levels must be tested to form a complete experimental matrix. Compared to single-factor experiments, the three-factor matrix experiment systematically explores all combinations of the three factors, comprehensively revealing main effects, interaction effects, and complex nonlinear relationships. The primary objective of this study is to analyze the influence patterns of impact parameter variations on the energy absorption performance of HGTCWs. Consequently, orthogonal array parameter selection for the three-factor experiment was designed to maximize coverage within feasible ranges, determined by the drop-weight machine’s maximum impact velocity, the heaviest available counterweight mass, and the load cell’s measurement capacity.

In impact scenarios, the impact energy is primarily determined by the mass and velocity of the impacting object. Therefore, variations in impact mass and velocity significantly influence structural damage, necessitating a detailed investigation into the specific effects of impact mass and velocity on the energy absorption performance of materials. In this study, the impact mass M was designed as 15 kg, 30 kg, and 45 kg, and the impact velocity V as 2 m/s, 3 m/s, and 4 m/s. Additionally, for HGTCWs, longer gradient heights result in greater material loss due to chemical etching, and increased etching areas lead to reduced stiffness. Thus, the etching height significantly affects the total mass and plastic deformation of honeycomb specimens. Based on this, the etching height H was selected as the third factor, with values of 50 mm, 100 mm, and 150 mm. The orthogonal experimental design matrix is shown in Table 2. Under identical impact conditions, 2–3 specimens were tested to ensure data repeatability and reliability.

## 3. Results and Discussion

### 3.1. Impact Compression Load–Displacement Curve Characteristics and Macroscopic Morphology of HGTCWs

**Figure 6:**
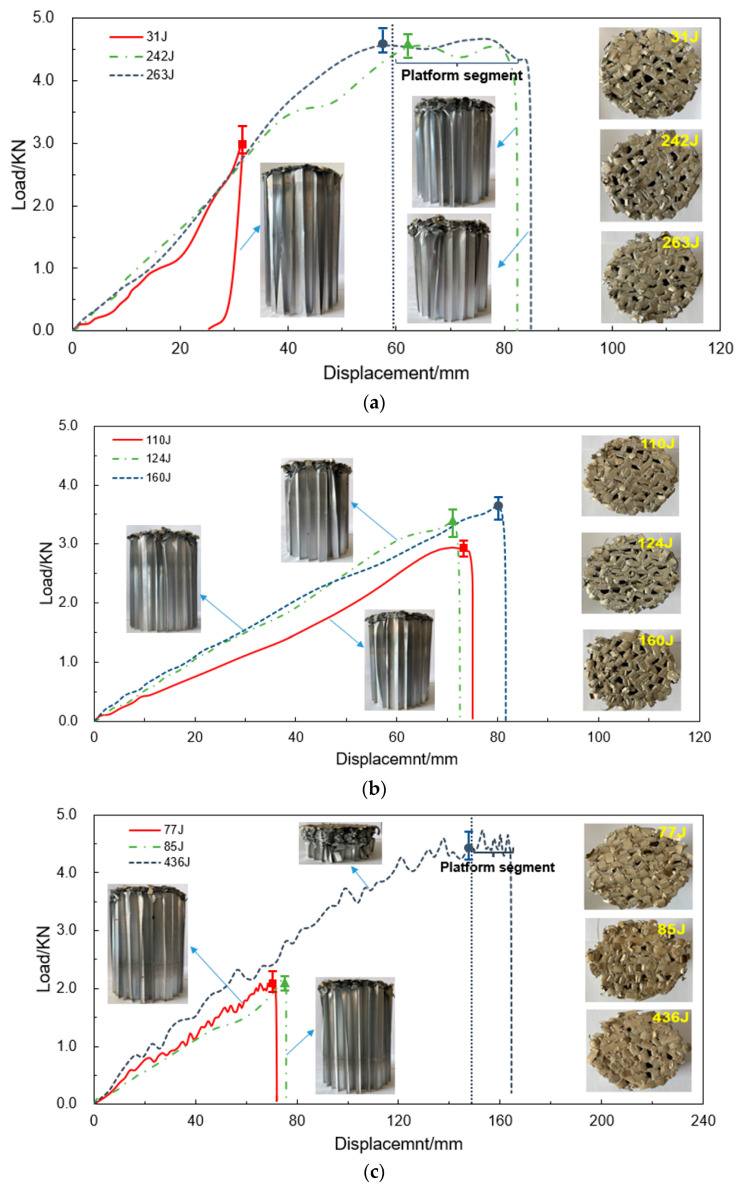
Load–displacement curves of aluminum HGTCWs under different impact conditions. (**a**) HGTCWs with an etching height of 50 mm. (**b**) HGTCWs with an etching height of 100 mm. (**c**) HGTCWs with an etching height of 150 mm.

It should be noted that the parameter settings for low-velocity impact testing in this study were constrained by the drop-weight machine’s maximum impact energy capacity while optimizing impact conditions across specimens. Due to limitations in the load cell’s measurement range, equipment protection protocols, and the orthogonal experiment’s focus on impact parameter analysis, specimens were not required to reach the densification stage typically observed in conventional impact tests. Figure 6 shows the load–displacement curves and post-impact macroscopic morphologies (side and impact surfaces) of HGTCW specimens with heights of 50 mm, 100 mm, and 150 mm under different impact conditions. As observed in Figure 6, none of the curves exhibit the transient peak load typically seen in traditional honeycomb materials during compression, demonstrating that linearly thinning the honeycomb walls through chemical etching completely eliminates peak loads.

Due to the significant height of the honeycomb specimens (200 mm), the gravitational potential energy corresponding to the drop height of the hammer from initial contact to the end of impact cannot be neglected. Therefore, the total absorbed energy should include both the initial impact kinetic energy (designed in Table 2) and the gravitational potential energy associated with the compression displacement. In Figure 6a, the load–displacement curves for the HGTCWs with an etching height of 50 mm (Experiments 1–3) exhibit nearly identical slopes, with stiffness values close to 0.083 kN/mm; the absorbed energies are 31 J, 242 J, and 263 J, respectively. In Experiment 1 (lowest impact mass and velocity), the compression displacement is less than 50 mm, and the load increases approximately linearly with displacement. Under the impact conditions of Experiments 2 and 3, after the compression displacement exceeds 50 mm, the load enters a plateau stage, where it oscillates slightly with increasing displacement, with a mean plateau load of approximately 4.55 kN.

In Figure 6b, for the HGTCWs with an etching height of 100 mm under the three impact conditions (Experiments 4–6), the specimens are not fully compressed in the graded-thickness region (compression displacements <100 mm); the load increases monotonically and linearly with displacement, reaching maximum values of 2.89 kN, 3.34 kN, and 3.58 kN, respectively. The stiffness under the impact condition of Experiment 4 is approximately 0.043 kN/mm, while in Experiments 5 and 6, the stiffness increases to 0.066 kN/mm. Correspondingly, the absorbed energy increases from 110 J (Experiment 4) to 124 J (Experiment 5) and 160 J (Experiment 6).

In Figure 6c, for the HGTCWs with an etching height of 150 mm in Experiments 7 and 8, the load–displacement curves still exhibit monotonic linear behavior, with closely matched material stiffness and maximum loads of approximately 0.034 kN/mm and 2.15 kN, respectively. The absorbed energies are 77 J and 85 J. However, in Experiment 9, when the compression displacement exceeds the etching height (150 mm), an inflection point appears in the load–displacement curve, marking the transition to a plateau phase. The material stiffness increases to 0.037 kN/mm, and the mean plateau load rises to 4.52 kN, resulting in a significant energy absorption increase to 436 J.

Post-impact macroscopic observations (Figure 6) reveal distinct morphological differences between HGTCWs and conventional HUCWs. In HGTCW specimens, the etched walls obscure most cellular pores after compression, unlike the preserved pore visibility in uniform-wall counterparts. This phenomenon arises from etching-induced defects and reduced stiffness in the graded region, which lead to weak initial load-bearing capacity and irregular plastic folding initiation at wall edges. Side-view images confirm periodic plastic folding patterns similar to uniform-wall honeycombs. Under the maximum impact velocity and mass conditions (Experiment 9), the honeycomb structure densifies into a nearly pore-free compacted layer with significantly increased material density.

In summary, the load–displacement characteristics of HGTCWs can be categorized into the following two types: (1) when the compressive displacement is less than the etching height, the curve remains in a single linear stage, where the load increases linearly with displacement. This reflects the fact that the structure is in the stage of uniform buckling deformation, indicating that the reduced stiffness of the etched regions in the HGTCW specimen transforms the contact between the drop-weight indenter and the specimen from “hard contact” (as in traditional uniform-thickness honeycombs) to “soft contact.” Consequently, the contact stress distribution becomes more uniform, and no significant local damage or stress mutation occurs in the specimen.

(2) When the compressive displacement exceeds the etching height, after the compressive displacement surpasses the etching height, the high-stiffness characteristics of the unetched regions begin to dominate the deformation, causing the load–displacement curve to enter the plateau stage. The load fluctuates slightly and remains nearly constant in this stage, which is dominated by the regular buckling and folding of the uniformly variable-thickness regions. Energy absorption enters a stable dissipation phase, with the specimen exhibiting a deformation mode where the etched regions collapse preferentially while the unetched regions provide auxiliary support. By continuously dissipating energy through controllable plastic deformation, this structure can avoid sudden failure. This characteristic endows it with significant application potential in impact resistance scenarios (e.g., collision protection), where the initiation point and duration of energy absorption can be regulated by designing the etching height.

### 3.2. Time–Velocity Curve Characteristics of HGTCWs Under Impact Compression

**Figure 7:**
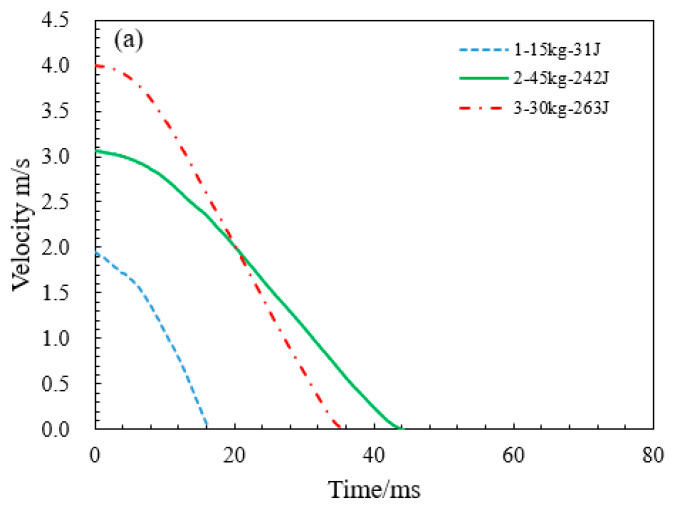

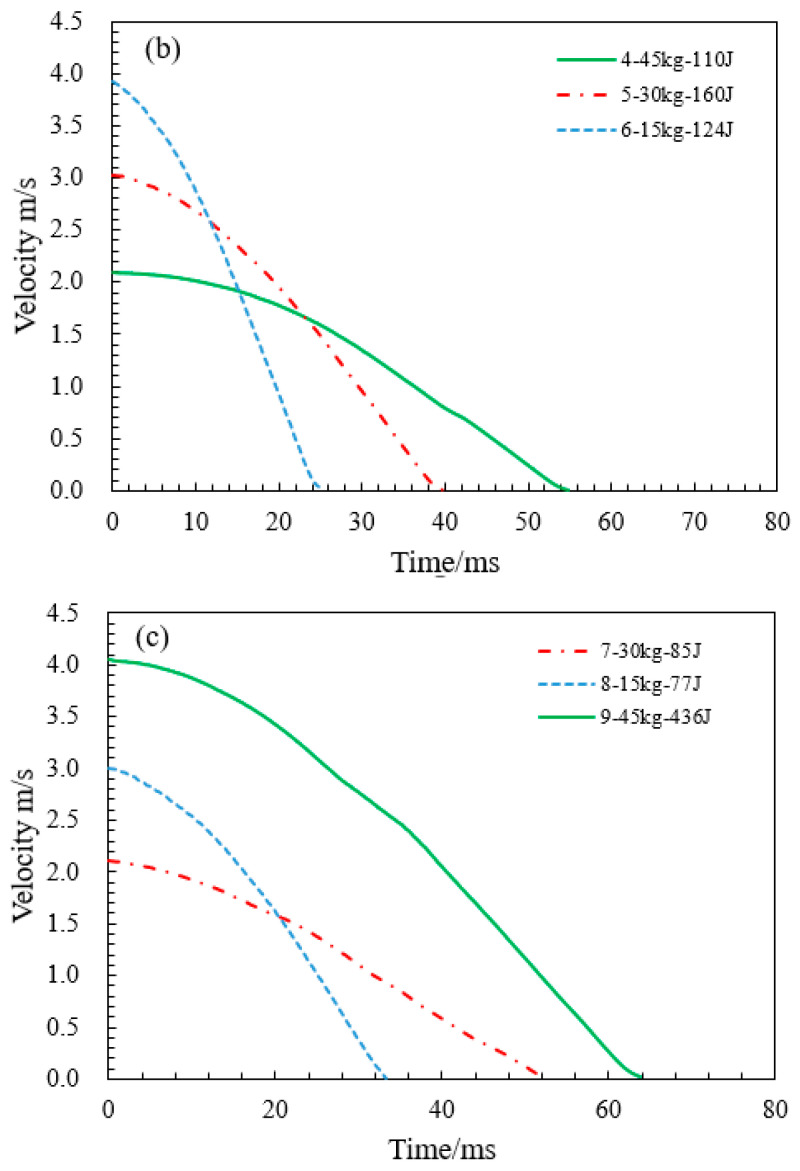
Time–velocity curves of HGTCWs under different impact conditions. (**a**) HGTCWs with an etching height of 50 mm. (**b**) HGTCWs with an etching height of 50 mm. (**c**) HGTCWs with an etching height of 50 mm.

The time–velocity curves during impact reveal the dynamic response of materials, providing critical insights into impact characteristics and energy absorption performance. Figure 7 compares the time–velocity profiles of HGTCWs across all orthogonal experimental conditions. As observed in Figure 7, regardless of impact conditions, the time–velocity curves consistently exhibit two distinct phases: an initial nonlinear deceleration phase followed by a linear deceleration phase. Differences in the shape of the curves are directly related to the coupling effects of impact mass, impact velocity, and etching height.

In Figure 7a, for the HGTCWs with an etching height of 50 mm, the impact durations in Experiments 2 and 3 are 44.1 ms and 35.4 ms, respectively, significantly longer than the 16.2 ms duration in Experiment 1. Notably, despite similar energy absorption levels in Experiments 2 and 3, higher impact masses correlate with prolonged impact durations and reduced slope magnitudes in the linear deceleration phase. Figure 7b demonstrates analogous trends for the HGTCWs with an etching height of 100 mm (Experiments 4–6). As the impact mass increases from 15 kg to 45 kg, the impact duration extends from 24.5 ms to 54.8 ms. This phenomenon primarily arises from enhanced inertial effects at higher masses, which prolong contact maintenance during compression, thereby extending energy dissipation. Similar curve characteristics and conclusions are observed in Figure 7c for the HGTCWs with an etching height of 150 mm under varying mass and velocity conditions.

Based on the variation patterns of the velocity–time curves from the matrix experiments, it can be concluded that the dominant role of etching height determines the “staged” degree of energy dissipation, and the curve’s descending rate can be controlled by adjusting the etching height; the primary influence of impact mass lies in regulating the momentum transfer rate—greater impact mass leads to a more gradual velocity decline. These conclusions provide guidance for parameter selection in engineering design. For example, if rapid energy dissipation is required in practical applications, HGTCWs with smaller etching heights should be chosen; if stable and slow energy absorption is needed, HGTCWs with larger etching heights should be selected. When the impact velocity is fixed, the energy absorption efficiency of the structure can be jointly regulated by adjusting the impact mass and etching height.

### 3.3. Three-Factor Matrix Experimental Data Analysis

One primary energy absorption mechanism of honeycomb materials involves progressive plastic folding deformation during impact [3], which prevents sudden energy release caused by abrupt structural failure. Consequently, the magnitude of plastic deformation serves as a critical metric for evaluating honeycomb performance. Specific energy absorption (*SEA*) defined as energy absorbed per unit mass (Formula (3)), is a core indicator for lightweight design [35]:

(3) $$SEA=\frac{E_{total}}{M}$$

where $E_{total}$ is the total absorbed energy and M the specimen mass. In the three-factor matrix experiments conducted in this study, since none of the specimens were fully compressed in the nine tests, Formula (4) was adopted for more accurate calculation of the specific energy absorption values of HGTCWs:

(4) $$SEA=\frac{E_{total}}{M_{L}}$$

where $M_{L}$ represents the mass of the plastically deformed region.

Plastic deformation ($P_{d}$), absorbed energy (E), specific energy absorption (SEA), and stiffness (k) were analyzed as key indicators. Table 3 summarizes the calculated values of these four reference indicators based on experimental data.

First, preliminary analysis is conducted based on the range R values. The range calculation formula is given in Equation (5). A larger range indicates that different levels of the factor have a greater influence on the results; thus, the magnitude of the range directly reflects the importance ranking of the factors.

(5) $$R_{i}={\bar{X}}_{imax}-{\bar{X}}_{imin}$$

where i represents the influencing factors (i.e., V, M, and H), and $X_{i}$ denotes the average value of the corresponding evaluation indicator for each factor. The calculation results are summarized in Table 4.

According to the calculation results in Table 4, the range value rankings of three performance evaluation indicators ($P_{d}$, E, and SEA) for HGTCWs are all $R_{V}$ > $R_{M}$ > $R_{H}$. This indicates that deformation displacement, energy absorption, and specific energy absorption are most significantly influenced by impact velocity. For honeycomb stiffness, the influence order is $R_{H}$ > $R_{V}\;$> $R_{M}$. This is primarily due to the increased thinning degree of honeycomb walls with elevated etching height, which naturally leads to reduced stiffness. Since range analysis does not account for experimental errors or interaction effects, merely reflecting differences in factor level means, it constitutes a qualitative analysis. Further quantitative analysis through analysis of variance is required to verify the significance of each factor. The degree of influence is determined by comparing the proportion of each factor’s sum of squares (SS) to the total sum of squares (SST). A larger SS value indicates greater variability caused by the factor, signifying stronger influence. The total sum of squares (SST), the sum of squares (${SS}_{i}$), the error sum of squares (${SS}_{e}$), and the mean square (${MS}_{i}$) for each factor are sequentially calculated using Equations (5)–(9):

(6) $$SST=\sum_{j=1}^{n}Y_{i}-\bar{Y}$$

Here, $Y_{j}$ refers to the three reference indicator values calculated from the data of nine orthogonal array experiments, i.e., the computed values in Table 3. $\bar{Y}\;$represents the grand mean. The value of SST primarily reflects the overall deviation of all data points from the grand mean.

(7) $${SS}_{i}=k\sum_{p=1}^{m}{\left({\bar{X}}_{i}-\bar{Y}\right)}^{2}$$

Here, k=3 represents the number of experimental runs per factor, and m=3 denotes the number of levels for each factor. For instance, the impact velocity factor includes three levels: 2 m/s, 3 m/s, and 4 m/s.

(8) $${SS}_{e}=SST-\left({SS}_{V}+{SS}_{M}+{SS}_{H}\right)$$

Before calculating the mean square (${MS}_{i}$), it is necessary to determine the degrees of freedom (df) in the analysis of variance. Since the degrees of freedom for each factor correspond to its number of levels minus 1, and all three factors in this study have three levels, each factor’s degree of freedom is 2. The total degrees of freedom (${df}_{total}$) are calculated as the total number of experimental runs minus 1, i.e., 9−1=8. The error degrees of freedom can then be derived from the following equation, yielding a value of 2.

(9) $${df}_{e}={df}_{total}-\left({df}_{V}+{df}_{M}+{df}_{H}\right)$$

(10) $${MS}_{i}=\frac{{SS}_{i}}{{df}_{i}}\;{MS}_{e}=\frac{{SS}_{e}}{{df}_{e}}$$

Finally, the significance of factor effects is evaluated by calculating the F-value, defined as the ratio of the factor mean square to the error mean square. A larger F-value indicates a more significant factor effect. Referring to the F-distribution table, at a significance level of α = 0.05 with degrees of freedom ${df}_{i}$ = 2 and ${df}_{e}=2$, the critical value $F_{0.05}\left(2,2\right)=19$. Thus, the significance criterion is as follows.

**Figure 8:**
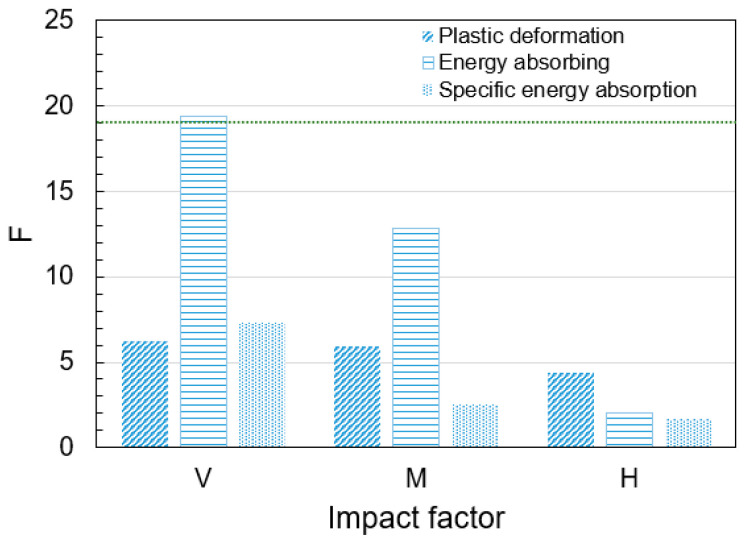
Comparison of F-value of three factors.

If $F_{i}>19$, the factor is significant; otherwise, it is not. The calculated values based on the above formulas are summarized in Table 5. Figure 8 compares the F-values of the three factors—impact velocity (V), impact mass (M), and etching height (H). As shown in Figure 8, the impact velocity factor exhibits the most significant effect on energy absorption (F>19), while the other factors are statistically insignificant. For plastic deformation of the HGTCWs, the effects of impact velocity and impact mass are comparable, exceeding the influence of etching height by approximately 36%. The impact velocity demonstrates a substantially stronger influence on energy absorption and specific energy absorption compared to variations in impact mass or etching height.

The ANOVA results align with the earlier range analysis, confirming the consistent significance ranking of factors: V>M>H. In studies on the impact compression performance of HGTCWs, it is recommended to prioritize optimization of impact velocity and mass, while the etching height can be selected based on practical requirements (e.g., lightweight design or manufacturing constraints). For instance, to reduce structural weight, increasing the etching height may enhance weight reduction, or to lower manufacturing costs, decreasing the etching height could shorten production cycles. This approach balances performance objectives with practical engineering considerations.

## 4. Conclusions

1. HUCWs often exhibit a high overload phenomenon in their load–displacement curves due to stress concentration caused by preferential collapse of cells at the impact end. In this study, the etching process with linearly decreasing cell wall thickness along the height direction successfully eliminated this peak load. By adjusting the etching height, the stiffness of the honeycomb can be effectively regulated, enabling HGTCWs to adapt to different operating conditions and application scenarios. For example, it can serve as a crash energy absorber for high-speed train noses or carriage connections. With its linearly tapering wall thickness design, energy is absorbed gradually along the honeycomb’s gradient direction during collisions, preventing sudden local failure and improving energy absorption efficiency. Alternatively, it can be used as a filler for automotive front longitudinal beams or bumper frames. During collisions, the etched thin-walled regions preferentially buckle to absorb energy, while the unetched thick-walled regions support the structure, enhancing safety performance.

Despite the advantages of HGTCWs in weight reduction and stiffness regulation, its dynamic responses (e.g., impact energy absorption, buckling modes) are influenced by the coupling effects of multiple factors, such as wall thickness gradient, cell size, and impact direction. Existing theoretical models for uniform honeycombs (e.g., constitutive relations based on homogenization assumptions) struggle to accurately describe the mechanical behavior of gradient structures. This requires extensive numerical simulations or experiments to refine the model, thereby increasing the design cycle of this type of honeycomb.

2. The load–displacement curve of the HGTCW exhibits a distinct bilinear characteristic, with its inflection point strictly aligned with the etching height, indicating that the etching height is a critical threshold governing the mechanical behavior of HGTCWs. When the compressive displacement is less than the etching height, the curve remains in a single linear stage, where the load increases linearly with displacement. When the compressive displacement exceeds the etching height, the curve enters a plateau stage, with minimal load fluctuations and near-constant values, indicating that the structure has transitioned to a stable energy absorption phase.

The bilinear characteristic of the time–velocity curve reflects the dynamic mechanism of deformation. The time–velocity curve is divided into an initial nonlinear descending segment and a linear descending segment, revealing the dynamic process of the HGTCW transitioning from “unstable initiation” to “stable energy absorption.” This feature provides a basis for predicting the velocity decay rate during impacts.

The influence of impact mass on the duration of energy absorption is related to structural inertia. Under conditions of comparable energy absorption, greater impact mass leads to longer impact durations. This indicates that the energy absorption process of HGTCWs is closely linked to the inertia of the impacting body: higher mass requires more time for the structure to dissipate energy through plastic deformation or collapse.

3. Range and variance analyses reveal that the sensitivity order of factors influencing the energy absorption characteristics of HGTCWs is impact velocity (v) > impact mass (m) > etching height (h). This conclusion provides clear parameter prioritization guidance for the practical engineering application of HGTCWs. Under resource constraints, the range analysis can quickly identify dominant factors, avoiding the resource waste of “full factorial experiments” and reducing research complexity. This helps engineers rapidly lock in key control variables under the constraints of “performance–cost–complexity,” minimizing design redundancy. Future research should further integrate machine learning or multi-physics coupled simulations to reveal the microscopic mechanisms underlying the influence of these factors—for example, how velocity affects cell wall deformation through strain rate hardening—to deepen the scientific understanding of the “v > m > h” sensitivity order.

## Figures and Tables

**Table 1:** Chemical composition of 5052 aluminum alloy foil (mass fraction/%) [31].

Si	Cu	Mg	Zn	Mn	Cr	Fe	Al
≤0.25	≤0.10	2.2~2.8	≤0.10	≤0.10	0.15~0.35	≤0.40	bal

**Table 2:** Three-factor orthogonal table.

Serial Number	Factor 1 Height of Thinning Wall (mm)	Factor 2 Velocity (m/s)	Factor 3 Weight of Impactor (kg)
1	50	2	15
2	50	3	45
3	50	4	30
4	100	2	45
5	100	3	30
6	100	4	15
7	150	2	30
8	150	3	15
9	150	4	45

**Table 3:** Calculated value of the reference indicators.

Serial Number	$P_{d}$	E (J)	SEA (J/g)	k (kN/mm)
1	0.16	31	7.14	0.079
2	0.39	242	15.66	0.083
3	0.425	263	15.34	0.083
4	0.365	110	8.70	0.043
5	0.41	124	5.88	0.066
6	0.36	160	14.12	0.066
7	0.375	85	6.73	0.034
8	0.36	77	6.54	0.037
9	0.81	436	15.23	0.037

**Table 4:** Analysis of range.

Factor	Numerical Value	${\bar{P}}_{d}$	$\bar{E}$ (J)	$\bar{SEA}$ (J/g)	$\bar{k}$ (kN/mm)
V	2 m/s	0.300	75.333	7.525	0.052
	3 m/s	0.387	147.667	9.364	0.062
	4 m/s	0.532	286.333	14.897	0.062
M	15 kg	0.293	89.333	9.268	0.061
	30 kg	0.403	157.333	9.318	0.061
	45 kg	0.522	262.667	3.932	0.054
H	50 mm	0.325	178.667	12.713	0.082
	100 mm	0.378	131.333	9.569	0.058
	150 mm	0.515	199.333	9.504	0.036
$R_{i}$	$R_{V}$	0.232	211.000	7.373	0.010
	$R_{M}$	0.228	173.333	3.932	0.007
	$R_{H}$	0.190	68.000	3.208	0.046

**Table 5:** Analysis of variance.

	$P_{d}$			E			SEA		
factor	${SS}_{i}$	${df}_{i}$	${MS}_{i}$	${SS}_{i}$	${df}_{i}$	${MS}_{i}$	${SS}_{i}$	${df}_{i}$	${MS}_{i}$
V	0.082	2	0.041	68,981.556	2	34,490.778	88.364	2	44.182
M	0.078	2	0.039	45,763.556	2	22,881.778	30.539	2	15.270
H	0.058	2	0.029	7291.556	2	3645.778	20.178	2	10.089
Error	0.013	2	0.007	3562.889	2	1781.444	12.122		6.061

## Data Availability

The raw data supporting the conclusions of this article will be made available by the authors on request.

## Abbreviations

The following abbreviations are used in this manuscript:

HGTCW	Honeycombs with Gradient-Thickness Cell Walls
HUCW	Honeycombs with Uniform Cell Walls
SEA	Specific Energy Absorption
SS	Sum of Squares
SST	Total Sum of Squares
MSi	Mean Square
df	Degrees of Freedom

## References

[1] Li S., Yang R., Sun S., Niu B. (2025). Advances in the analysis of honeycomb structures: A comprehensive review. Compos. Part B-Eng..

[2] Wu Y., Fang J., He Y., Li W. (2018). Crashworthiness of hierarchical circular-joint quadrangular honeycombs. Thin-Wall. Struct..

[3] Smahat A., Mankour A., Slimane S., Roubache R., Bendine K., Guelailia A. (2020). Numerical investigation of debris impact on spacecraft structure at hyper-high velocity. J. Braz. Soc. Mech. Sci. Eng..

[4] Davies G., Hitchings D., Besant T., Clarke A., Morgan C. (2004). Compression after impact strength of composite sandwich panels. Compos. Struct..

[5] Sanchez-Saez S., Barbero E., Zaera R., Navarro C. (2005). Compression after impact of thin composite laminates. Compos. Sci. Technol..

[6] Daniel I.M., Abot J.L., Schubel P.M., Luo J. (2012). Response and damage tolerance of composite sandwich structures under low velocity impact. Exp. Mech..

[7] Wei Y., Zhang Y., Song Q., Zhou X., Zhou Y., Shen Y. (2022). Effects of different configurations and gradients on compression responses of gradient honeycombs via selective laser melting. Thin-Wall. Struct..

[8] Nazir A., Arshad A.B., Lin S.C., Jeng J.Y. (2022). Mechanical performance of lightweight designed honeycomb structures fabricated using multijet fusion additive manufacturing technology. 3D Print. Addit. Manuf..

[9] Pirouzfar S., Zeinedini A. (2021). Effect of geometrical parameters on the flexural properties of sandwich structures with 3D-printed honeycomb core and E-glass/epoxy Face sheets. Structures.

[10] Zhou H., Fang H., Wu H., Xu J. (2023). Effects of inter-cell connections on the multi-stable dynamics of dual-cell stacked Miura-origami structures. J. Sound Vib..

[11] Li Z., Wen Y., Wen X., Hao H., Chen W. (2023). Single and double-layered kirigami corrugated sandwich panels against impact loads. Structures.

[12] Cheng-Hsin C., Jong-Shin H. (2002). Effects of solid distribution on the elastic buckling of honeycombs. Int. J. Mech. Sci..

[13] Cheng-Hsin C., Jong-Shin H. (2002). Elastic moduli and plastic collapse strength of hexagonal honeycombs with plateau borders. Int. J. Mech. Sci..

[14] Ma J., Hou D., Chen Y., You Z. (2016). Quasi-static axial crushing of thin-walled tubes with a kite-shape rigid origami pattern: Numerical simulation. Thin-Wall. Struct..

[15] Albert P.C., Ghani A., Othman M.Z., Zaidi A. (2016). Axial crushing behavior of aluminum square tube with origami pattern. Mod. Appl. Sci..

[16] Zhai J., Liu Y., Geng X., Zheng W., Zhao Z., Cui C., Li M. (2019). Energy absorption of pre folded honeycomb under in-plane dynamic loading. Thin-Wall. Struct..

[17] Townsend S., Adams R., Robinson M., Hanna B., Theobald P. (2020). 3D printed origami honeycombs with tailored out-of-plane energy absorption behavior. Mater. Des..

[18] Li Z., Yang Q., Fang R., Chen W., Hao H. (2021). Origami metamaterial with two-stage programmable compressive strength under quasi-static loading. Int. J. Mech. Sci..

[19] Li Q., Zhi X., Fan F. (2022). Dynamic crushing of uniform and functionally graded origami inspired cellular structure fabricated by SLM. Eng. Struct..

[20] Zhai J., Zhang D., Li M., Cui C., Cai J. (2022). Out-of-plane energy absorption and crush behavior of origami honeycomb. Thin-Wall. Struct..

[21] Wang S., Pei W., Yang Y., Ding Y. (2023). Design and analysis of buckling-induced honeycombs with tailorable out-of-plane crushing performance. Aerosp. Sci. Technol..

[22] Francesco T., Matteo V., Rossana D., Maria A. (2021). Higher order formulations for doubly-curved shell structures with a honeycomb core. Thin-Wall. Struct..

[23] Gibson L., Ashby M., Schajer G. (1982). The mechanics of two-dimensional cellular materials. Proc. R. Soc. London Ser. A Math. Phys. Eng. Sci..

[24] Valentin M., Tan S., Yicha Z., Nadia L., François P. (2024). Multiscale periodic homogenization for additive manufacturing of honeycomb lattices. Int. J. Solids Struct..

[25] Mukhopadhyay T., Adhikari S., Batou A. (2019). Frequency domain homogenization for the viscoelastic properties of spatially correlated quasi-periodic lattices. Int. J. Mech. Sci..

[26] Geers M., Kouznetsova V. (2010). Multi-scale computational homogenization: Trends and challenges. J. Comput. Appl. Math..

[27] Nady K., Dos Reis F., Ganghoffer J. (2017). Computation of the homogenized nonlinear elastic response of 2D and 3D auxetic structures based on micropolar continuum models. Compos. Struct..

[28] Kumar A., Muthu N., Narayanan R. (2023). Equivalent orthotropic properties of periodic honeycomb structure: Strain-energy approach and homogenization. Int. J. Mech. Mater. Des..

[29] Mohammad K., Zeinab S., Ehsan A., Luca L., Civalek Ö. (2021). Thermo-elastic buckling of honeycomb micro plates integrated with FG-GNPs reinforced Epoxy skins with stretching effect. Compos. Struct..

[30] Hossein A., Mohammad K., Zeinab S., Rossana D., Francesco T. (2020). Quasi-3D hyperbolic shear deformation theory for the free vibration study of honeycomb microplates with graphene nanoplatelets-reinforced epoxy skins. Molecule.

[31] Singh L., Singh A., Singh B. (2022). MAG shield gas influence study on impact strength of Al-5052 joint. Mater. Today.

[32] Xu S., Beynon J.H., Ruan D., Yu T.X. (2019). Strength enhancement of aluminium honeycombs caused by entrapped air under dynamic out-of-plane compression. Int. J. Impact Eng..

[33] Manikandan P., Chai G.B. (2015). A similitude approach towards the understanding of low velocity impact characteristics of bi-layered hybrid composite structures. Compos. Struct..

[34] Zhang Y., Zhu W., Chu X., Shi L., Zhan X., Cheng H., Sun L. (2025). The mechanical behavior of designing recycled hot-mix asphalt containing fine RAP particles with multiple parameters using orthogonal experimental approach. Constr. Build. Mater..

[35] Seyed Yaghoubi A., Liaw B. (2012). Thickness influence on ballistic impact behaviors of GLARE 5 fiber-metal laminated beams: Experimental and numerical studies. Compos. Struct..

